# The genome sequence of the Piedmont Ringlet, *Erebia meolans* von Prunner, 1798 (Lepidoptera: Nymphalidae)

**DOI:** 10.12688/wellcomeopenres.24665.1

**Published:** 2025-08-01

**Authors:** Marta Vila, Kay Lucek, Charlotte J. Wright, Joana I. Meier, Mark L. Blaxter, Wellcome Sanger Institute Tree of Life Management, Samples and Laboratory team, Wellcome Sanger Institute Scientific Operations: Sequencing Operations, Wellcome Sanger Institute Tree of Life Core Informatics team, Tree of Life Core Informatics collective, Project Psyche Community

**Affiliations:** 1 Universidade da Coruna, A Coruña, Galicia, Spain; 2 University of Neuchâtel, Neuchâtel, Switzerland; 3 Tree of Life, Wellcome Sanger Institute, Hinxton, England, UK

**Keywords:** Erebia meolans, Piedmont Ringlet, genome sequence, chromosomal, Lepidoptera

## Abstract

We present a genome assembly from a female specimen of *Erebia meolans* (Piedmont Ringlet; Arthropoda; Insecta; Lepidoptera; Nymphalidae). The assembly contains two haplotypes with total lengths of 512.88 megabases and 439.35 megabases. Most of haplotype 1 (95.0%) is scaffolded into 15 chromosomal pseudomolecules, including the W and Z sex chromosomes. Haplotype 2 was assembled to scaffold level. The mitochondrial genome has also been assembled, with a length of 15.24 kilobases.

## Species taxonomy

Eukaryota; Opisthokonta; Metazoa; Eumetazoa; Bilateria; Protostomia; Ecdysozoa; Panarthropoda; Arthropoda; Mandibulata; Pancrustacea; Hexapoda; Insecta; Dicondylia; Pterygota; Neoptera; Endopterygota; Amphiesmenoptera; Lepidoptera; Glossata; Neolepidoptera; Heteroneura; Ditrysia; Obtectomera; Papilionoidea; Nymphalidae; Satyrinae; Erebiini; *Erebia*; *Erebia meolans* von Prunner, 1798 (NCBI:txid111904)

## Background

*Erebia meolans*, commonly known as the Piedmont Ringlet, is a univoltine butterfly species belonging to the family Nymphalidae (subfamily Satyrinae). This medium-sized butterfly is characterised by its dark brown forewings, which feature a distinctive orange postdiscal band containing up to five eyespots, and a variable dorsal hindwing pattern. Females are generally larger and exhibit more prominent eyespots.

The size and wing markings exhibit considerable variability. For instance, specimens of the subspecies *E. m. bejarensis* (Chapman, 1902) from central Spain are the largest in wingspan and exhibit a strongly defined wing pattern ( Higgins, 1975; Tolman & Lewington, 2008). The high intraspecific variability in the colouration of *E. meolans* can complicate its identification, leading to confusion with similar species. These include its sister species *E. palarica*, which is generally larger in areas of sympatry ( Torrado-Blanco, 2024), and *E. triaria*, especially when the characteristic S6 eyespot is absent and flight periods coincide.

*Erebia meolans* is widely distributed across mountainous regions of central and southwestern Europe, including the ones in the northern half of the Iberian Peninsula, the Alps, the Apennines, and the French Massif Central. Populations are usually found at altitudes between 300 and 2 000 metres. Habitats include steep slopes, montane grasslands, meadows, and forest clearings. The role of *E. meolans* in montane ecosystems both as a pollinator and a prey species underscores its ecological importance.

Caterpillars of *E. meolans* feed on various grasses of the genera *Festuca* and *Poa*, among others (see Clarke, 2024 for a comprehensive list of host plants). The morphological description of the different larval stages has been reviewed by García-Barros & Fartmann (2009), e.g. showing chromatic dimorphism in their last instar (see also Sonderegger, 2005). The species completes its life cycle with overwintering as larvae and pupation occurring in the ground, among plant debris.

Although *E. meolans* is not currently under significant threat and is categorised as Least Concern (LC) by the IUCN ( van Swaay *et al.*, 2010), its sensitivity to ecological pressures, particularly those arising from climate change, warrants attention. Indeed, *E. meolans* has experienced local extinctions in several areas (e.g. in the Thuringian Forest, Germany, extinct before 1980, T. Schmitt pers. comm.) and it is considered a vulnerable species in Austria ( Huemer *et al.*, 2022).

The chromosome level reference genome of *E. meolans* not only confirms the chromosome number inferred by de Lesse (1960), but also offers valuable opportunities to study adaptation to montane habitats and ecological resilience. Furthermore, it paves the way for comparative studies between closely related species, enhancing our understanding of evolutionary processes and informing conservation strategies for montane butterflies.

## Methods

### Sample acquisition

**Figure 1.**
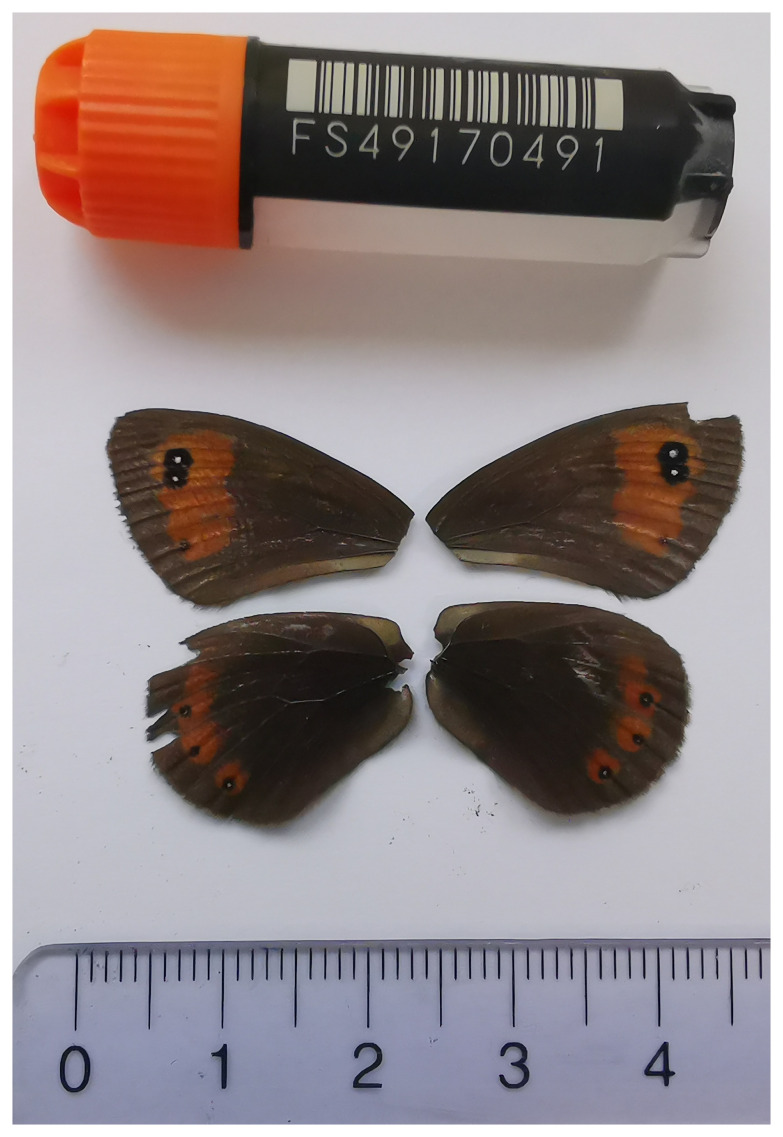
Voucher photograph of the *Erebia meolans* (ilEreMeol1) specimen used for genome sequencing.

The specimen used for genome sequencing was an adult female *Erebia meolans* (specimen ID SAN28000020, ToLID ilEreMeol1; Figure 1), collected from Finhaut, Valais, Switzerland (latitude 46.0875, longitude 6.9801; elevation 1 375 m) on 17/06/2023. The specimen was collected and identified by Kay Lucek (University of Neuchâtel).

### Nucleic acid extraction

Protocols for high molecular weight (HMW) DNA extraction developed at the Wellcome Sanger Institute (WSI) Tree of Life Core Laboratory are available on protocols.io ( Howard *et al.*, 2025). The ilEreMeol1 sample was weighed and triaged to determine the appropriate extraction protocol. Tissue from the thorax was homogenised by powermashing using a PowerMasher II tissue disruptor.

HMW DNA was extracted in the WSI Scientific Operations core using the Automated MagAttract v2 protocol. DNA was sheared into an average fragment size of 12–20 kb following the Megaruptor®3 for LI PacBio protocol. Sheared DNA was purified by automated SPRI (solid-phase reversible immobilisation). The concentration of the sheared and purified DNA was assessed using a Nanodrop spectrophotometer and Qubit Fluorometer using the Qubit dsDNA High Sensitivity Assay kit. Fragment size distribution was evaluated by running the sample on the FemtoPulse system. For this sample, the final post-shearing DNA had a Qubit concentration of 34.18 ng/μL and a yield of 1 606.46 ng, with a fragment size of 16.0 kb. The 260/280 spectrophotometric ratio was 1.95, and the 260/230 ratio was 2.66.

### PacBio HiFi library preparation and sequencing

Library preparation and sequencing were performed at the WSI Scientific Operations core. Libraries were prepared using the SMRTbell Prep Kit 3.0 (Pacific Biosciences, California, USA), following the manufacturer’s instructions. The kit includes reagents for end repair/A-tailing, adapter ligation, post-ligation SMRTbell bead clean-up, and nuclease treatment. Size selection and clean-up were performed using diluted AMPure PB beads (Pacific Biosciences). DNA concentration was quantified using a Qubit Fluorometer v4.0 (ThermoFisher Scientific) and the Qubit 1X dsDNA HS assay kit. Final library fragment size was assessed with the Agilent Femto Pulse Automated Pulsed Field CE Instrument (Agilent Technologies) using the gDNA 55 kb BAC analysis kit.

The sample was sequenced on a Revio instrument (Pacific Biosciences). The prepared library was normalised to 2 nM, and 15 μL was used for making complexes. Primers were annealed and polymerases bound to generate circularised complexes, following the manufacturer’s instructions. Complexes were purified using 1.2X SMRTbell beads, then diluted to the Revio loading concentration (200–300 pM) and spiked with a Revio sequencing internal control. The sample was sequenced on a Revio 25M SMRT cell. The SMRT Link software (Pacific Biosciences), a web-based workflow manager, was used to configure and monitor the run and to carry out primary and secondary data analysis.

Specimen details, sequencing platforms, and data yields are summarised in Table 1.

**Table 1.** Specimen and sequencing data for BioProject PRJEB78678.

Platform	PacBio HiFi	Hi-C
**ToLID**	ilEreMeol1	ilEreMeol1
**Specimen ID**	SAN28000020	SAN28000020
**BioSample (source individual)**	SAMEA115109515	SAMEA115109515
**BioSample (tissue)**	SAMEA115109639	SAMEA115109645
**Tissue**	thorax	head
**Sequencing platform and model**	Revio	Illumina NovaSeq X
**Run accessions**	ERR13485710	ERR13474175
**Read count total**	2.02 million	658.60 million
**Base count total**	23.11 Gb	99.45 Gb

### Hi-C

***Sample preparation and crosslinking***

The Hi-C sample was prepared from 20–50 mg of frozen head tissue of the ilEreMeol1 sample using the Arima-HiC v2 kit (Arima Genomics). Following the manufacturer’s instructions, tissue was fixed and DNA crosslinked using TC buffer to a final formaldehyde concentration of 2%. The tissue was homogenised using the Diagnocine Power Masher-II. Crosslinked DNA was digested with a restriction enzyme master mix, biotinylated, and ligated. Clean-up was performed with SPRISelect beads before library preparation. DNA concentration was measured with the Qubit Fluorometer (Thermo Fisher Scientific) and Qubit HS Assay Kit. The biotinylation percentage was estimated using the Arima-HiC v2 QC beads.

***Hi-C library preparation and sequencing***

Biotinylated DNA constructs were fragmented using a Covaris E220 sonicator and size selected to 400–600 bp using SPRISelect beads. DNA was enriched with Arima-HiC v2 kit Enrichment beads. End repair, A-tailing, and adapter ligation were carried out with the NEBNext Ultra II DNA Library Prep Kit (New England Biolabs), following a modified protocol where library preparation occurs while DNA remains bound to the Enrichment beads. Library amplification was performed using KAPA HiFi HotStart mix and a custom Unique Dual Index (UDI) barcode set (Integrated DNA Technologies). Depending on sample concentration and biotinylation percentage determined at the crosslinking stage, libraries were amplified with 10–16 PCR cycles. Post-PCR clean-up was performed with SPRISelect beads. Libraries were quantified using the AccuClear Ultra High Sensitivity dsDNA Standards Assay Kit (Biotium) and a FLUOstar Omega plate reader (BMG Labtech).

Prior to sequencing, libraries were normalised to 10 ng/μL. Normalised libraries were quantified again and equimolar and/or weighted 2.8 nM pools. Pool concentrations were checked using the Agilent 4200 TapeStation (Agilent) with High Sensitivity D500 reagents before sequencing. Sequencing was performed using paired-end 150 bp reads on the Illumina NovaSeq X.

Specimen details, sequencing platforms, and data yields are summarised in Table 1.

### Genome assembly

Prior to assembly of the PacBio HiFi reads, a database of *k*-mer counts ( *k* = 31) was generated from the filtered reads using FastK. GenomeScope2 ( Ranallo-Benavidez *et al.*, 2020) was used to analyse the *k*-mer frequency distributions, providing estimates of genome size, heterozygosity, and repeat content.

The HiFi reads were assembled using Hifiasm in Hi-C phasing mode ( Cheng *et al.*, 2021; Cheng *et al.*, 2022), producing two haplotypes. Hi-C reads ( Rao *et al.*, 2014) were mapped to the primary contigs using bwa-mem2 ( Vasimuddin *et al.*, 2019). Contigs were further scaffolded with Hi-C data in YaHS ( Zhou *et al.*, 2023), using the --break option for handling potential misassemblies. The scaffolded assemblies were evaluated using Gfastats ( Formenti *et al.*, 2022), BUSCO ( Manni *et al.*, 2021) and MERQURY.FK ( Rhie *et al.*, 2020).

The mitochondrial genome was assembled using MitoHiFi ( Uliano-Silva *et al.*, 2023), which runs MitoFinder ( Allio *et al.*, 2020) and uses these annotations to select the final mitochondrial contig and to ensure the general quality of the sequence.

### Assembly curation

The assembly was decontaminated using the Assembly Screen for Cobionts and Contaminants ( ASCC) pipeline. TreeVal was used to generate the flat files and maps for use in curation. Manual curation was conducted primarily in PretextView and HiGlass ( Kerpedjiev *et al.*, 2018). Scaffolds were visually inspected and corrected as described by Howe *et al*. (2021). Manual corrections included 14 breaks and 52 joins. The curation process is documented at https://gitlab.com/wtsi-grit/rapid-curation. PretextSnapshot was used to generate a Hi-C contact map of the final assembly.

### Assembly quality assessment

Chromosomal painting was performed using lep_busco_painter using Merian elements, which represent the 32 ancestral linkage groups in Lepidoptera ( Wright *et al.*, 2024). Painting was based on gene locations from the lepidoptera_odb10 BUSCO analysis and chromosome lengths from the genome index produced using SAMtools faidx ( Danecek *et al.*, 2021). Each complete BUSCO (including both single-copy and duplicated BUSCOs) was assigned to a Merian element using a reference database, and coloured positions were plotted along chromosomes drawn to scale.

The Merqury.FK tool ( Rhie *et al.*, 2020), run in a Singularity container ( Kurtzer *et al.*, 2017), was used to evaluate *k*-mer completeness and assembly quality for both haplotypes using the *k*-mer databases ( *k* = 31) computed prior to genome assembly. The analysis outputs included assembly QV scores and completeness statistics.

The genome was analysed using the BlobToolKit pipeline, a Nextflow implementation of the earlier Snakemake BlobToolKit pipeline ( Challis *et al.*, 2020). The pipeline aligns PacBio reads using minimap2 ( Li, 2018) and SAMtools ( Danecek *et al.*, 2021) to generate coverage tracks. Simultaneously, it queries the GoaT database ( Challis *et al.*, 2023) to identify relevant BUSCO lineages and runs BUSCO ( Manni *et al.*, 2021). For the three domain-level BUSCO lineages, BUSCO genes are aligned to the UniProt Reference Proteomes database ( Bateman *et al.*, 2023) using DIAMOND blastp ( Buchfink *et al.*, 2021). The genome is divided into chunks based on the density of BUSCO genes from the closest taxonomic lineage, and each chunk is aligned to the UniProt Reference Proteomes database with DIAMOND blastx. Sequences without hits are chunked using seqtk and aligned to the NT database with blastn ( Altschul *et al.*, 1990). The BlobToolKit suite consolidates all outputs into a blobdir for visualisation. The BlobToolKit pipeline was developed using nf-core tooling ( Ewels *et al.*, 2020) and MultiQC ( Ewels *et al.*, 2016), with package management via Conda and Bioconda ( Grüning *et al.*, 2018), and containerisation through Docker ( Merkel, 2014) and Singularity ( Kurtzer *et al.*, 2017).

## Genome sequence report

### Sequence data

The genome of a specimen of *Erebia meolans* was sequenced using Pacific Biosciences single-molecule HiFi long reads, generating 23.11 Gb (gigabases) from 2.02 million reads, which were used to assemble the genome. GenomeScope2.0 analysis estimated the haploid genome size at 473.16 Mb, with a heterozygosity of 1.14% and repeat content of 31.10%. These estimates guided expectations for the assembly. Based on the estimated genome size, the sequencing data provided approximately 47× coverage. Hi-C sequencing produced 99.45 Gb from 658.60 million reads, which were used to scaffold the assembly. Table 1 summarises the specimen and sequencing details.

### Assembly statistics

The genome was assembled into two haplotypes using Hi-C phasing. Haplotype 1 was curated to chromosome level, while haplotype 2 was assembled to scaffold level. The final assembly has a total length of 512.88 Mb in 90 scaffolds, with 108 gaps, and a scaffold N50 of 35.84 Mb ( Table 2).

**Table 2.** Genome assembly statistics.

**Assembly name**	ilEreMeol1.hap1.1	ilEreMeol1.hap2.1
**Assembly accession**	GCA_964271795.1	GCA_964271775.1
**Assembly level**	chromosome	scaffold
**Span (Mb)**	512.88	439.35
**Number of chromosomes**	15	N/A
**Number of contigs**	198	111
**Contig N50**	8.08 Mb	9.04 Mb
**Number of scaffolds**	90	19
**Scaffold N50**	35.84 Mb	35.82 Mb
**Longest scaffold length (Mb)**	56.87	N/A
**Sex chromosomes**	W and Z	N/A
**Organelles**	Mitochondrial genome: 15.24 kb	N/A

**Figure 2.**
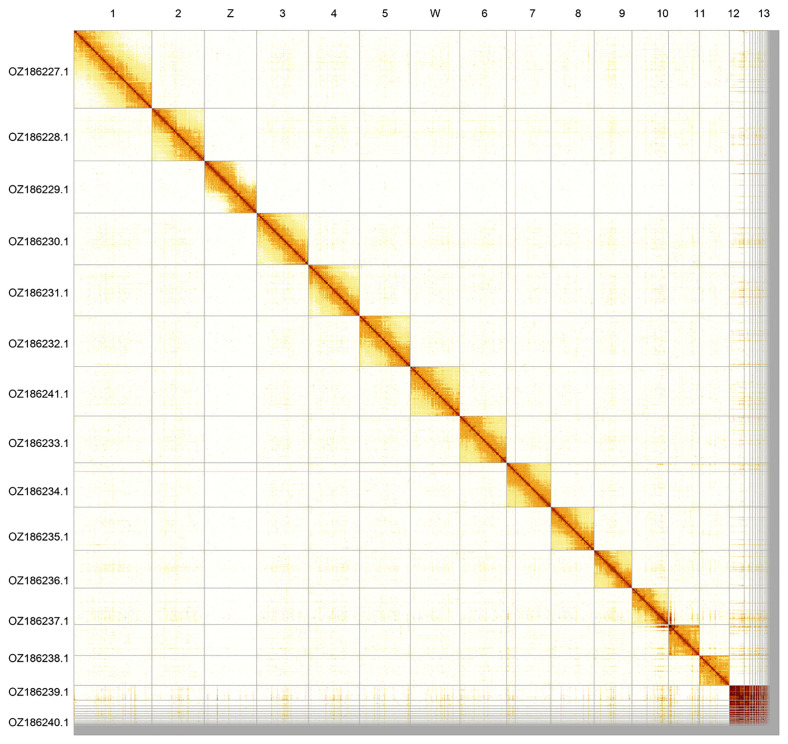
Hi-C contact map of the *Erebia meolans* genome assembly. Assembled chromosomes are shown in order of size and labelled along the axes. The plot was generated using PretextSnapshot.

**Figure 3.**
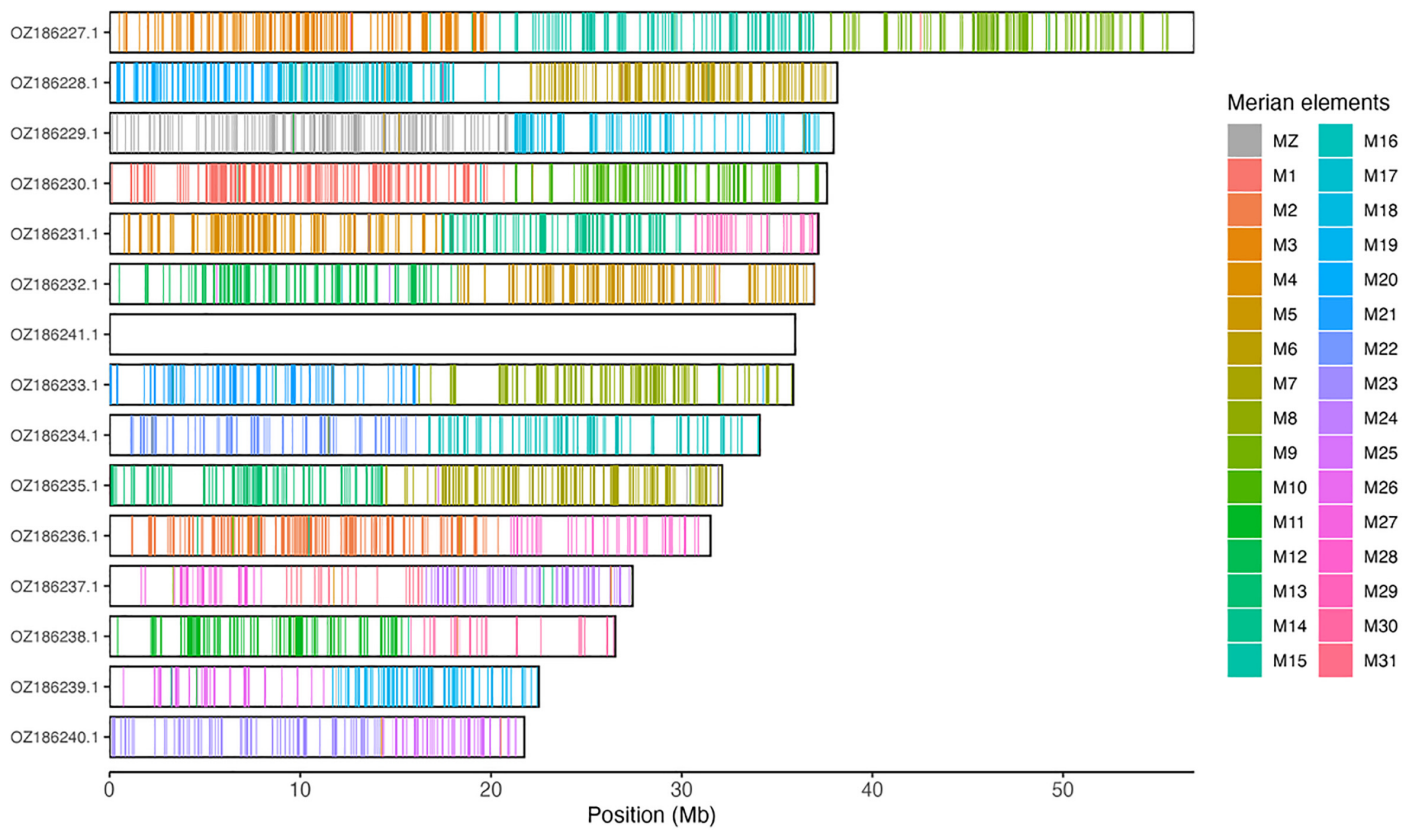
Merian elements painted across chromosomes in the ilEreMeol1.hap1.1 assembly of *Erebia meolans*. Chromosomes are drawn to scale, with the positions of orthologues shown as coloured bars. Each orthologue is coloured by the Merian element that it belongs to. All orthologues which could be assigned to Merian elements are shown.

Most of the assembly sequence (95.0%) was assigned to 15 chromosomal-level scaffolds, representing 13 autosomes and the W and Z sex chromosomes. The sex chromosomes were identified by read coverage. These chromosome-level scaffolds, confirmed by Hi-C data, are named according to size ( Figure 2; Table 3). Chromosome painting with Merian elements illustrates the distribution of orthologues along chromosomes and highlights patterns of chromosomal evolution relative to Lepidopteran ancestral linkage groups ( Figure 3).

**Table 3.** Chromosomal pseudomolecules in the haplotype 1 genome assembly of *Erebia meolans* ilEreMeol1.

INSDC accession	Molecule	Length (Mb)	GC%	Assigned Merian elements
OZ186227.1	1	56.87	37	M15;M3;M9
OZ186228.1	2	38.16	37	M17;M20;M6
OZ186230.1	3	37.62	37	M1;M10
OZ186231.1	4	37.17	37	M14;M29;M4
OZ186232.1	5	36.95	37	M12;M5
OZ186233.1	6	35.84	37	M21;M8
OZ186234.1	7	34.09	37	M16;M22
OZ186235.1	8	32.11	37	M13;M7
OZ186236.1	9	31.51	37	M2;M28
OZ186237.1	10	27.42	37	M24;M27;M31
OZ186238.1	11	26.52	37.50	M11;M30
OZ186239.1	12	22.51	37.50	M19;M26
OZ186240.1	13	21.75	37.50	M23;M25
OZ186241.1	W	35.95	40	N/A
OZ186229.1	Z	37.96	36.50	M18;MZ

The mitochondrial genome was also assembled. This sequence is included as a contig in the multifasta file of the genome submission and as a standalone record.

### Assembly quality metrics

**Figure 4.**
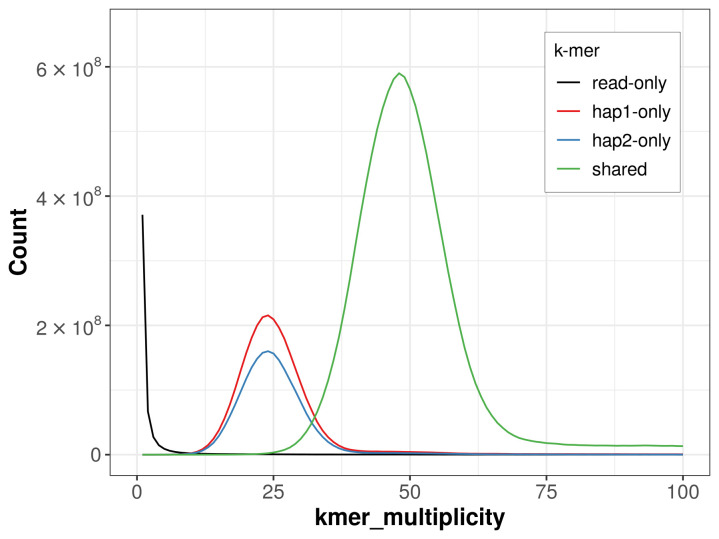
Evaluation of *k*-mer completeness using MerquryFK. This plot illustrates the recovery of *k*-mers from the original read data in the final assemblies. The horizontal axis represents *k*-mer multiplicity, and the vertical axis shows the number of *k*-mers. The black curve represents *k*-mers that appear in the reads but are not assembled. The green curve (the homozygous peak) corresponds to *k*-mers shared by both haplotypes and the red and blue curves (the heterozygous peaks) show *k*-mers found only in one of the haplotypes.

**Figure 5.**
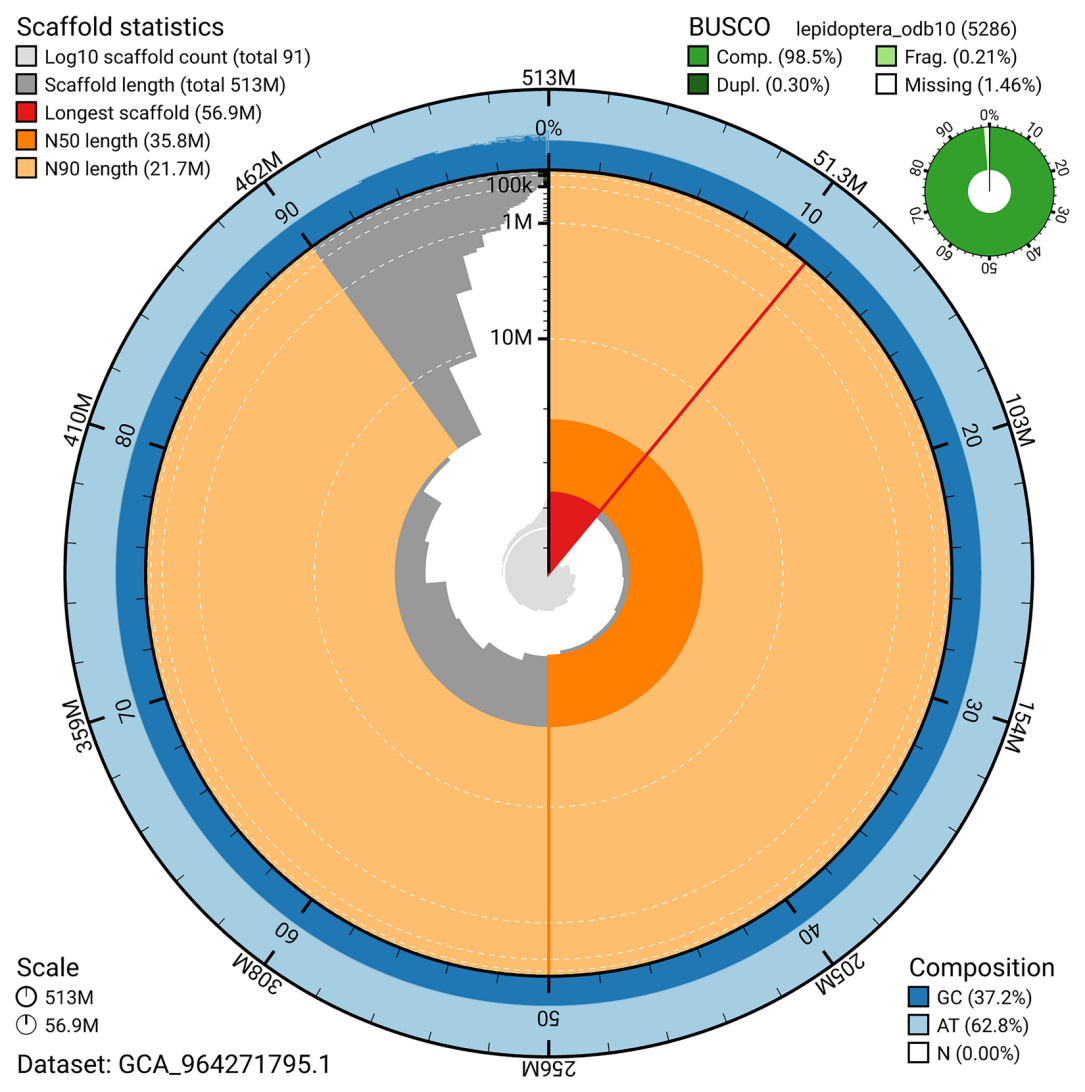
Assembly metrics for ilEreMeol1.hap1.1. The BlobToolKit snail plot provides an overview of assembly metrics and BUSCO gene completeness. The circumference represents the length of the whole genome sequence, and the main plot is divided into 1,000 bins around the circumference. The outermost blue tracks display the distribution of GC, AT, and N percentages across the bins. Scaffolds are arranged clockwise from longest to shortest and are depicted in dark grey. The longest scaffold is indicated by the red arc, and the deeper orange and pale orange arcs represent the N50 and N90 lengths. A light grey spiral at the centre shows the cumulative scaffold count on a logarithmic scale. A summary of complete, fragmented, duplicated, and missing BUSCO genes in the set is presented at the top right. An interactive version of this figure can be accessed on the BlobToolKit viewer.

**Figure 6.**
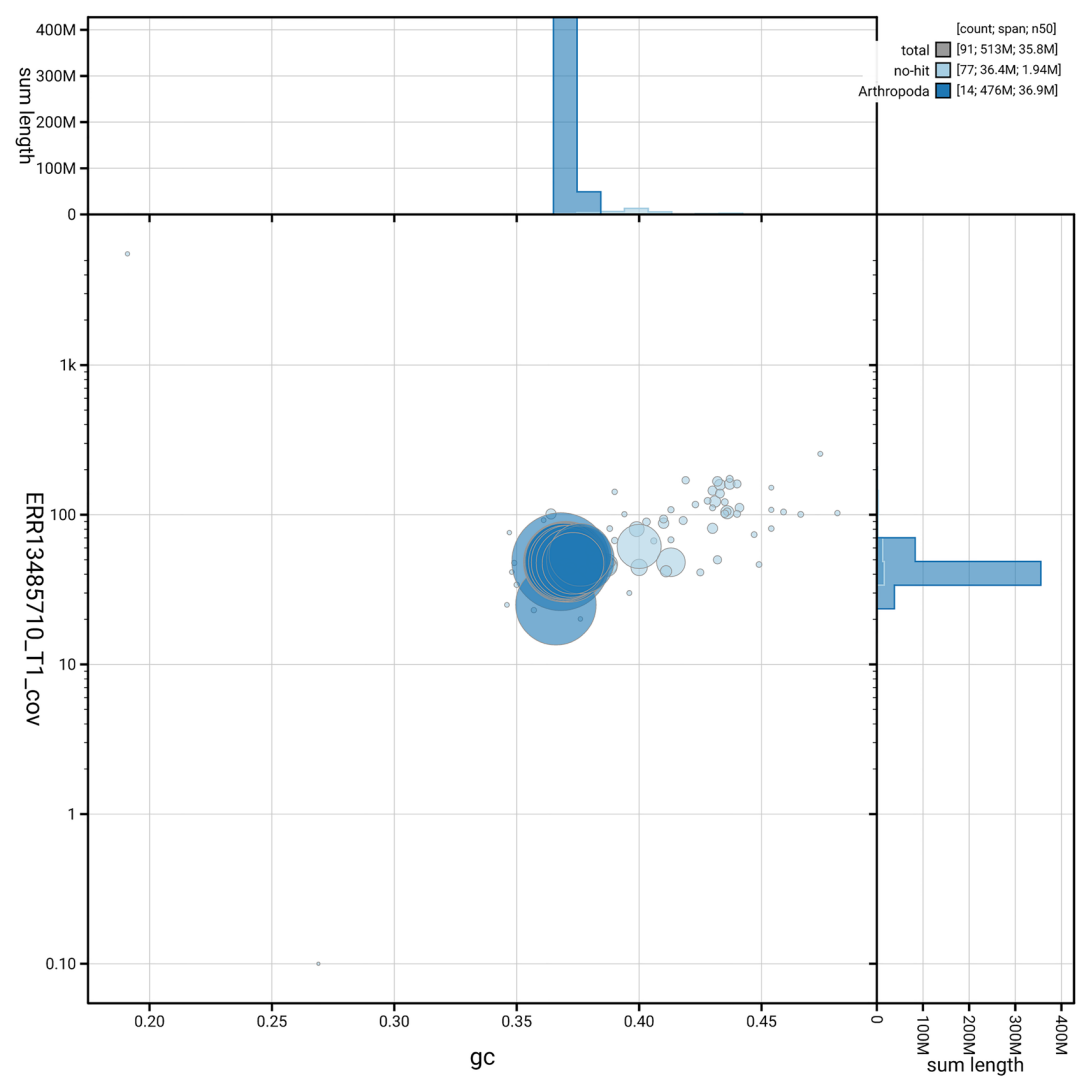
BlobToolKit GC-coverage plot for ilEreMeol1.hap1.1. Blob plot showing sequence coverage (vertical axis) and GC content (horizontal axis). The circles represent scaffolds, with the size proportional to scaffold length and the colour representing phylum membership. The histograms along the axes display the total length of sequences distributed across different levels of coverage and GC content. An interactive version of this figure is available on the BlobToolKit viewer.

For haplotype 1, the estimated QV is 65.6, and for haplotype 2, 65.3. When the two haplotypes are combined, the assembly achieves an estimated QV of 65.4. The *k*-mer completeness is 80.08% for haplotype 1, 73.26% for haplotype 2, and 99.41% for the combined haplotypes ( Figure 4). BUSCO analysis using the lepidoptera_odb10 reference set ( *n* = 5 286) ( Kriventseva *et al.*, 2019) identified 98.5% of the expected gene set (single = 98.2%, duplicated = 0.3%) for haplotype 1. The snail plot in Figure 5 summarises the scaffold length distribution and other assembly statistics for haplotype 1. The blob plot in Figure 6 shows the distribution of scaffolds by GC proportion and coverage for haplotype 1.

Table 4 lists the assembly metric benchmarks adapted from Rhie *et al.* (2021) the Earth BioGenome Project Report on Assembly Standards September 2024. The EBP metric, calculated for the haplotype 1, is **6.C.Q65**, meeting the recommended reference standard.

**Table 4.** Earth Biogenome Project summary metrics for the *Erebia meolans* assembly.

Measure	Value	(Benchmark)
EBP summary (haplotype 1)	6.C.Q65	6.C.Q40
Contig N50 length	8.08 Mb	≥ 1 Mb
Scaffold N50 length	35.84 Mb	= chromosome N50
Consensus quality (QV)	Haplotype 1: 65.6; haplotype 2: 65.3; combined: 65.4	≥ 40
*k*-mer completeness	Haplotype 1: 80.08%; Haplotype 2: 73.26%; combined: 99.41%	≥ 95%
BUSCO	C:98.5%[S:98.2%‚D:0.3%]‚ F:0.2%‚M:1.2%‚n:5 286	S > 90%; D < 5%
Percentage of assembly assigned to chromosomes	95%	≥ 90%

### Wellcome Sanger Institute – Legal and Governance

The materials that have contributed to this genome note have been supplied by a Tree of Life collaborator. The Wellcome Sanger Institute employs a process whereby due diligence is carried out proportionate to the nature of the materials themselves, and the circumstances under which they have been/are to be collected and provided for use. The purpose of this is to address and mitigate any potential legal and/or ethical implications of receipt and use of the materials as part of the research project, and to ensure that in doing so, we align with best practice wherever possible. The overarching areas of consideration are:

- Ethical review of provenance and sourcing of the material
- Legality of collection, transfer and use (national and international).

Each transfer of samples is undertaken according to a Research Collaboration Agreement or Material Transfer Agreement entered into by the Tree of Life collaborator, Genome Research Limited (operating as the Wellcome Sanger Institute), and in some circumstances, other Tree of Life collaborators.

## Data Availability

European Nucleotide Archive: Erebia meolans (piedmont ringlet). Accession number PRJEB78678. The genome sequence is released openly for reuse. The *Erebia meolans* genome sequencing initiative is part of the Sanger Institute Tree of Life Programme (PRJEB43745) and Project Psyche (PRJEB71705). All raw sequence data and the assembly have been deposited in INSDC databases. The genome will be annotated using available RNA-Seq data and presented through Ensembl at the European Bioinformatics Institute. Raw data and assembly accession identifiers are reported in Table 1 and Table 2. Pipelines used for genome assembly at the WSI Tree of Life are available at https://pipelines.tol.sanger.ac.uk/pipelines. Table 5 lists software versions used in this study.
