# Supplementary material for: The genome sequence of the Piedmont Ringlet, Erebia meolans von Prunner, 1798 (Lepidoptera: Nymphalidae)
Source: Wellcome Open Res. 2025 Aug 1;10:408. [Version 1] doi: 10.12688/wellcomeopenres.24665.1 (PMC12475900; doi:10.12688/wellcomeopenres.24665.1)
Supplement: Peer review history

# Reviewer report: Reviewer response for version 1

**DOI:** 10.21956/wellcomeopenres.27180.r130729

**Published:** 2025-09-26

**Authors:** Sivasankaran Kuppusamy

**Affiliations:** 1 Loyola College, Chennai, India

A comprehensive chromosome-level genome of *Erebia meolans* von Pruner, 1798 was sequenced, assembled and annotated using appropriate methods and softwares. The assembly contains two haplotypes with lengths of 512.88 Mb and 439.35 Mb, respectively. Through scaffolding authors have detected 15 chromosomal pseudomolecules.  

 Comments on the Manuscript

 The sentence of the background can change as *Erebia meolans*, von Prunner, 1798 commonly known as the ………………………

 “Specimen details, sequencing platforms, and data yields are summarized in Table 1”. The caption was used two times in the text. One can be deleted.

 Throughout the text, the author consistently used the genus' full name *Erebia meolans*. Start with the full genus name once, if possible, then shorten it to *E. meolans*.

 Sequence annotation details like number of protein-coding genes Gene transcripts, non-coding genes have not been given in the text and also not given the annotation table.

 The research article was well prepared and the manuscript meets the necessary scientific standard and is suitable for indexing.

Are sufficient details of methods and materials provided to allow replication by others?

Yes

Is the rationale for creating the dataset(s) clearly described?

Yes

Are the datasets clearly presented in a useable and accessible format?

Yes

Are the protocols appropriate and is the work technically sound?

Yes

Reviewer Expertise:

Phylogenetic analysis of Noctuoidea moths using mitogenome sequence

I confirm that I have read this submission and believe that I have an appropriate level of expertise to confirm that it is of an acceptable scientific standard.

# Reviewer report: Reviewer response for version 1

**DOI:** 10.21956/wellcomeopenres.27180.r130727

**Published:** 2025-08-27

**Authors:** Stephan Koblmüller

**Affiliations:** 1 University of Graz, Graz, Austria

This manuscript by Vila et al. presents the chromosomal-level genome assembly of *Erebia meolans, a nymphalid butterfly* , widely distributed in the mountains of central and soutwestern Europe. The authors provide comprehensive species background information and detailed methodologies covering specimen collection, sequencing, assembly, and quality assessments. I have no major concerns but have one minor comment:

 1. Background: "The morphological description of the different larval stages has been reviewed by García-Barros & Fartmann (2009), e.g. showing chromatic dimorphism in their last instar (see also Sonderegger, 2005)" - please rephrase. As it reads now, the sentence implies the the morphological descriptiton shows chromatic dimorphism in the last instar ...

Are sufficient details of methods and materials provided to allow replication by others?

Yes

Is the rationale for creating the dataset(s) clearly described?

Yes

Are the datasets clearly presented in a useable and accessible format?

Yes

Are the protocols appropriate and is the work technically sound?

Yes

Reviewer Expertise:

phylogenetics/-genomics, population genetics, phylogeography

I confirm that I have read this submission and believe that I have an appropriate level of expertise to confirm that it is of an acceptable scientific standard.
